# Supplementary material for: Fully inkjet-printed Ag2Se flexible thermoelectric devices for sustainable power generation
Source: Nat Commun. 2024 Mar 8;15:2141. doi: 10.1038/s41467-024-46183-1 (PMC10923913; doi:10.1038/s41467-024-46183-1)
Supplement: Supplementary file 1 — Supplementary Information [file 41467_2024_46183_MOESM1_ESM.pdf]

Supplementary information

Fully inkjet-printed Ag_2Se flexible thermoelectric devices for sustainable power generation

Yan Liu, Qihao Zhang, Aibin Huang, Keyi Zhang, Shun Wan, Hongyi Chen, Yuntian Fu, Wusheng Zuo, Yongzhe Wang, Xun Cao, Lianjun Wang, Uli Lemmer, and Wan Jiang

Y. Liu, K. Y. Zhang, Y. T. Fu, W. S. Zuo, Prof. L. J. Wang, Prof. W. Jiang
State Key Laboratory for Modification of Chemical Fibers and Polymer, Materials & College of Materials Science and Engineering, Donghua University, Shanghai, 201620, China.
E-mail: wanglj@dhu.edu.cn, wanjiang@dhu.edu.cn

Dr. Q. H. Zhang, Prof. Uli Lemmer
Light Technology Institute, Karlsruhe Institute of Technology, Engesserstrasse 13, 76131 Karlsruhe, Germany.
Email: qihao.zhang@kit.edu

Prof. A. B. Huang, Dr. Y. Z. Wang, Prof. X. Cao
State Key Laboratory of High Performance Ceramics and Superfine Microstructure, Shanghai Institute of Ceramics, Chinese Academy of Sciences, Shanghai, 200050, China.
Email: cxun@mail.sic.ac.cn

Dr. S. Wan
Center for High Pressure Science and Technology Advanced Research (HPSTAR), Shanghai, 201203, China.

Prof. H. Y. Chen
College of Chemistry and Chemical Engineering, Central South University, Changsha, 410083, China.

Prof. L. J. Wang
Engineering Research Center of Advanced Glasses Manufacturing Technology, Ministry of Education, Donghua University, Shanghai, 201620, China.

Prof. Uli Lemmer
Institute of Microstructure Technology (IMT), Karlsruhe Institute of Technology (KIT), Hermann-von-Helmholtz-Platz 1, 76344 Eggenstein-Leopoldshafen, Germany.

Prof. W. Jiang
Institute of Functional Materials, Donghua University, Shanghai, 201620, China.

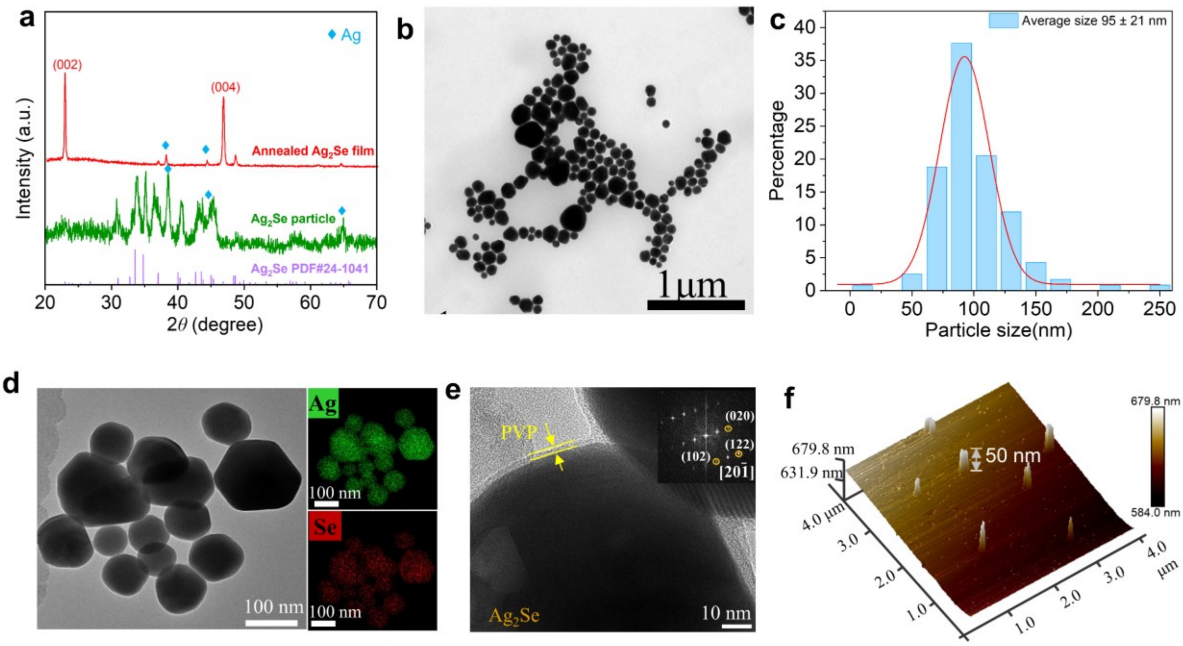

Supplementary Figure 1| Microstructural characterization of as-synthesized Ag_2Se nanoparticles. **a,** Powder X-ray diffraction (XRD) patterns. **b,** Low-magnification transmission electron microscopy (TEM) image of Ag_2Se nanoparticles and (**c**) corresponding average particle size distribution. **d,** TEM image and corresponding EDS elemental mapping results. **e,** High-resolution TEM image with the inset showing the corresponding FFT patterns. **f,** Atomic force microscopy (AFM) image and corresponding height profile. The uncertainty in the thickness value determined by AFM is ±5 nm.

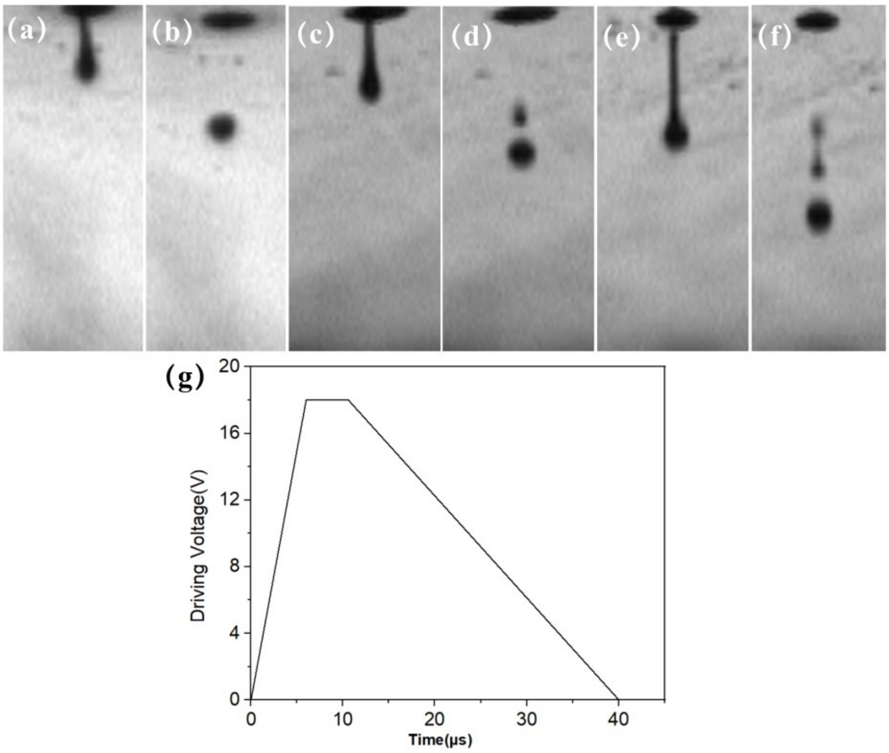

Supplementary Figure 2| Shape of droplets when using different cartridge voltage (**a**) and (**b**) 18 V, (**c**) and (**d**) 21 V, (e) and (**f**) 26 V. **g.** Jetting waveform used in printing Ag_2Se-based inks.

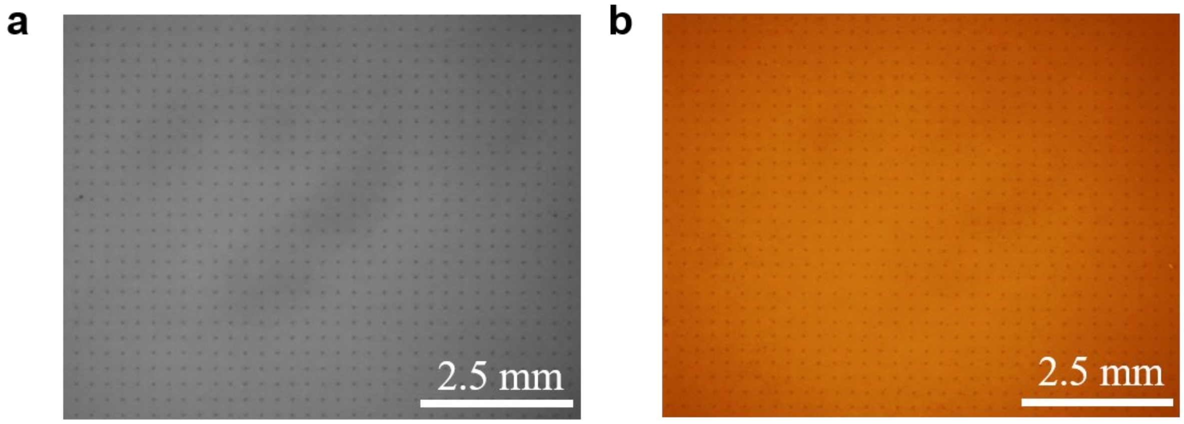

Supplementary Figure 3| Inkjet-printed Ag_2Se droplets on (**a**) photographic paper for better visibility and (**b**) polyimide substrate.

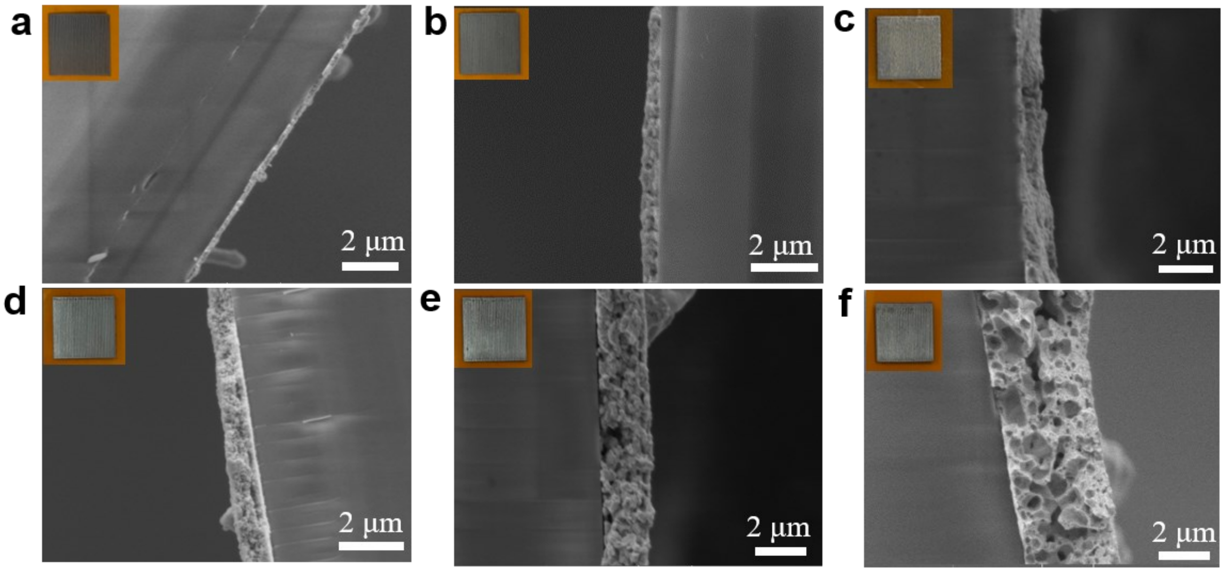

Supplementary Figure 4| Cross-sectional SEM images of printed Ag_2Se film with different printing layers and the inset shows the optical photographs of corresponding films. **a**, 10 layers. **b**, 20 layers. **c**, 30 layers. **d**, 40 layers. **e**, 60 layers. **f**, 80 layers.

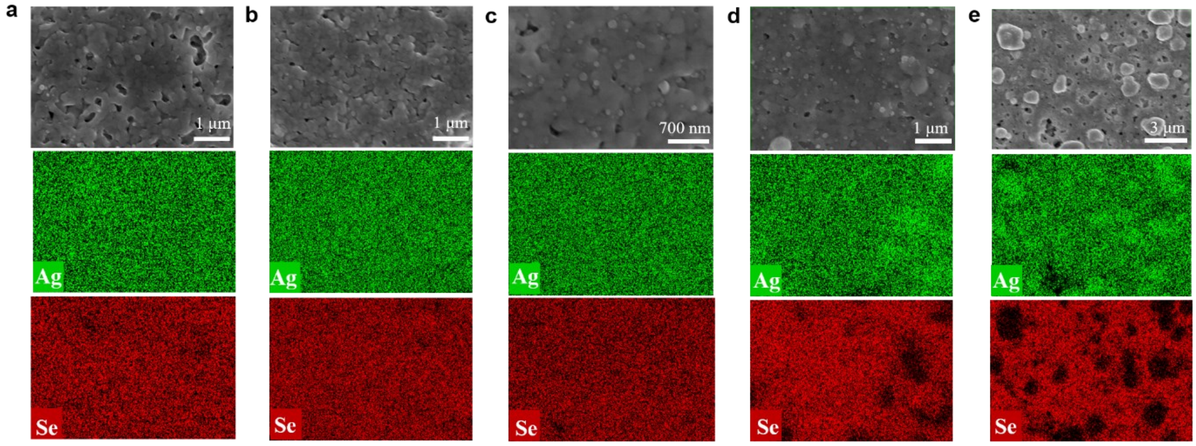

Supplementary Figure 5| SEM and corresponding EDS mapping of annealed films. a, Ag_2Se. **b**, Ag_2Se/10%Ag. **c**, Ag_2Se/15%Ag. **d**, Ag_2Se/20%Ag. **e**, Ag_2Se/25%Ag.

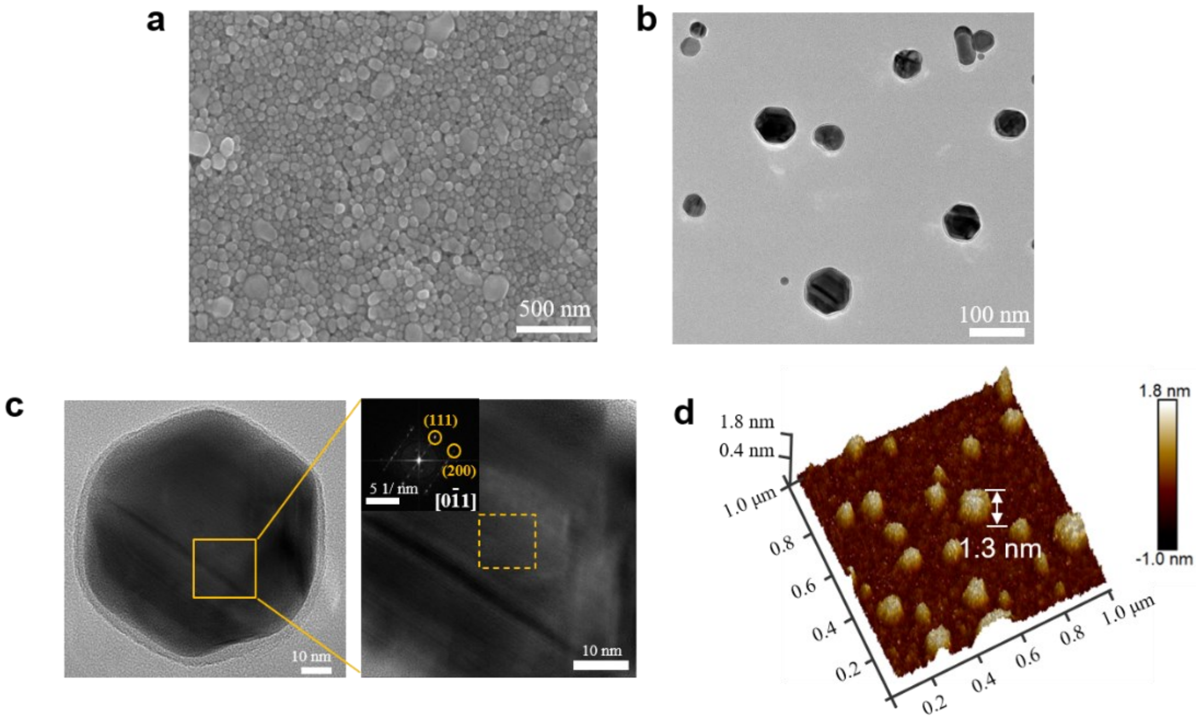

Supplementary Figure 6| Microstructural characterization of commercially available Ag ink for inkjet printing. a, Scanning electron microscopy (SEM) image, showing Ag nano-particles with the size of around 100 nm. **b,** Transmission electron microscopy (TEM) image. **c,** High-resolution TEM image with the corresponding FFT pattern, corresponding to cubic Ag. **d,** AFM image and corresponding height profile.

SEM and TEM images indicate that the Ag nanoparticles have a narrow size distribution and an average lateral diameter of approximately 100 nm (**Supplementary Fig. 6a and 6b**). High-resolution TEM image shows lattice spacing of 2.4 Å, corresponding to the (111) lattice plane of cubic Ag, as confirmed by fast Fourier transform (FFT) pattern (**Supplementary Fig. 6c**). In addition, atomic force microscopy (AFM) analysis estimates that the average thickness of the Ag particles is 1.3 nm (**Supplementary Fig. 6d**).

Supplementary Table 1| Inverse Ohnesorge number *Z* of Ag_2Se-based inks.

Ink	Density ($g \cdot cm^{-3}$)	Viscosity ($mPa \cdot s$)	Surface tensity ($mN \cdot m^{-1}$)	Concentration ($mg \cdot mL^{-1}$)	*Z*
Ag_2Se	0.794	1.41	21.75	10	13.5
Ag_2Se/15%Ag	0.796	1.52	24.28	10	13.3

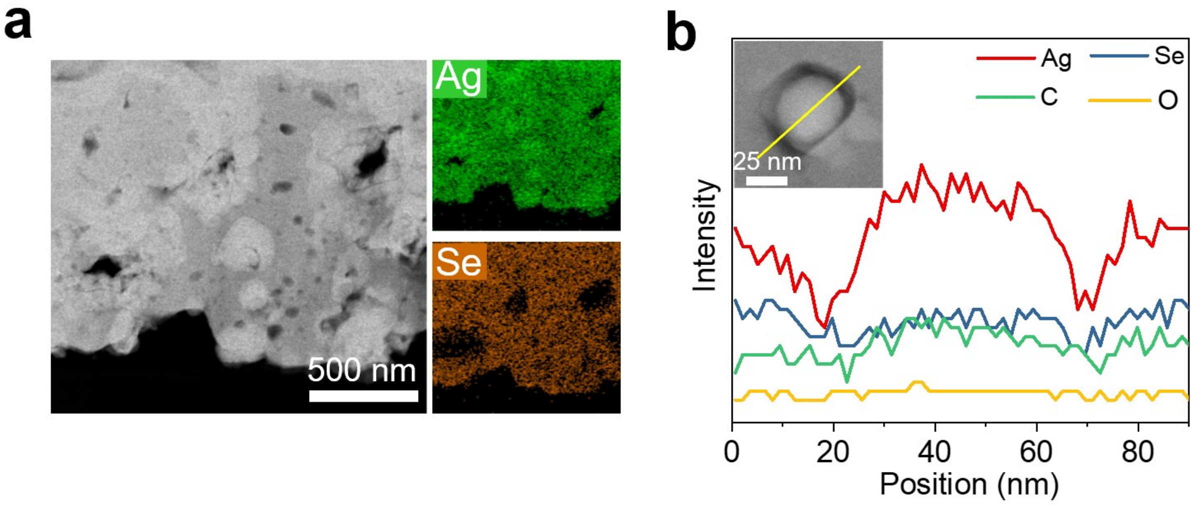

Supplementary Figure 7| a, HAADF-STEM image of the cross section of Ag_2Se/15%Ag composite film, and corresponding EDS elemental mapping. **b,** EDS line scan performed along the region marked in the inset, showing the variation of elements across the Ag_2Se and Ag interface.

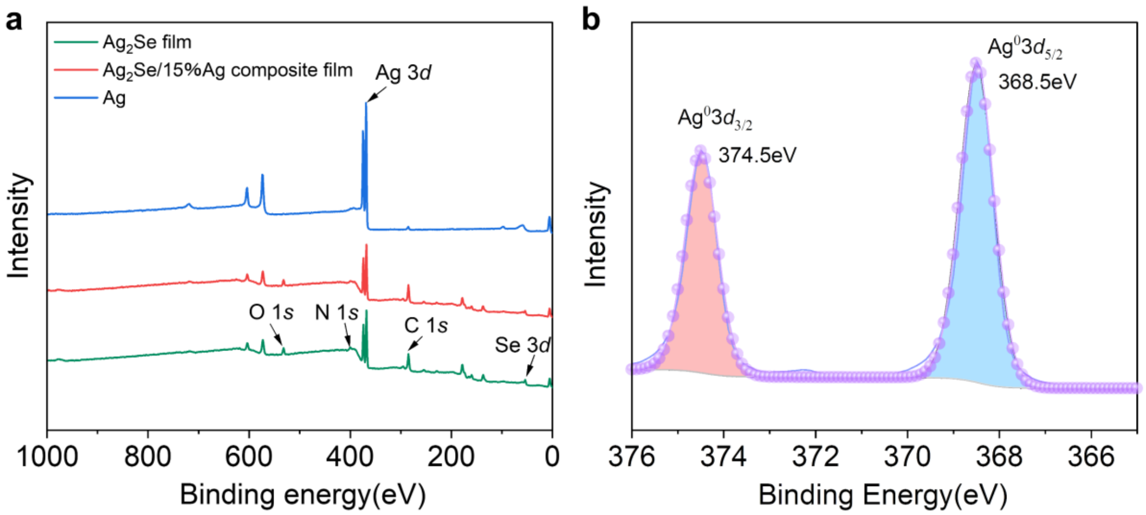

Supplementary Figure 8| a, XPS spectrum of Ag_2Se-based films and Ag ink. **b**, High-resolution data of Ag 3*d* XPS spectra of Ag ink.

Table S2. Comparison of atomic percentage of all elements according to XPS results.

Compound	Ag 3*d*	C 1*s*	O 1*s*	N 1*s*	Se 3*d*
Ag_2Se	11.92%	59.89%	11.17%	6.76%	10.25%
Ag_2Se-10%Ag	7.86%	66.91%	9.93%	8.92%	6.38%
Ag_2Se-15%Ag	12.04%	61.05%	11.42%	7.09%	8.40%
Ag_2Se-20%Ag	8.58%	64.11%	12.81%	9.16%	5.34%
Ag_2Se-25%Ag	10.77%	60.38%	10.91%	13.57%	4.38%

Table S3. Peak binding energies.

Compound	Ag $3d_{3/2}$ Binding energy, eV		Ag $3d_{5/2}$ Binding energy, eV		Binding energy, eV	
	Ag^0 $3d_{3/2}$	Ag^+ $3d_{3/2}$	Ag^0 $3d_{5/2}$	Ag^+ $3d_{5/2}$	Se $3d_{3/2}$	Se $3d_{5/2}$
Ag_2Se	374.49	373.98	368.53	368	54.56	53.68
Ag_2Se-10%Ag	374.49	374.05	368.50	368.09	54.47	53.62
Ag_2Se-15%Ag	374.46	374.06	368.51	368.08	54.62	53.74
Ag_2Se-20%Ag	374.55	374.1	368.57	368.13	54.49	53.61
Ag_2Se-25%Ag	374.51	374.13	368.54	368.11	54.52	53.58

Table S4. Atomic percentages from the analysis of high-resolution XPS and Ag^0/Ag^+ ratio data.

Sample	Ag/Se ratio by XPS	Ag^0/Ag^+ (%)
Ag_2Se	1.16	19.83
Ag_2Se-10%Ag	1.23	25.22
Ag_2Se-15%Ag	1.43	30.69
Ag_2Se-20%Ag	1.61	38.94
Ag_2Se-25%Ag	2.46	45.48

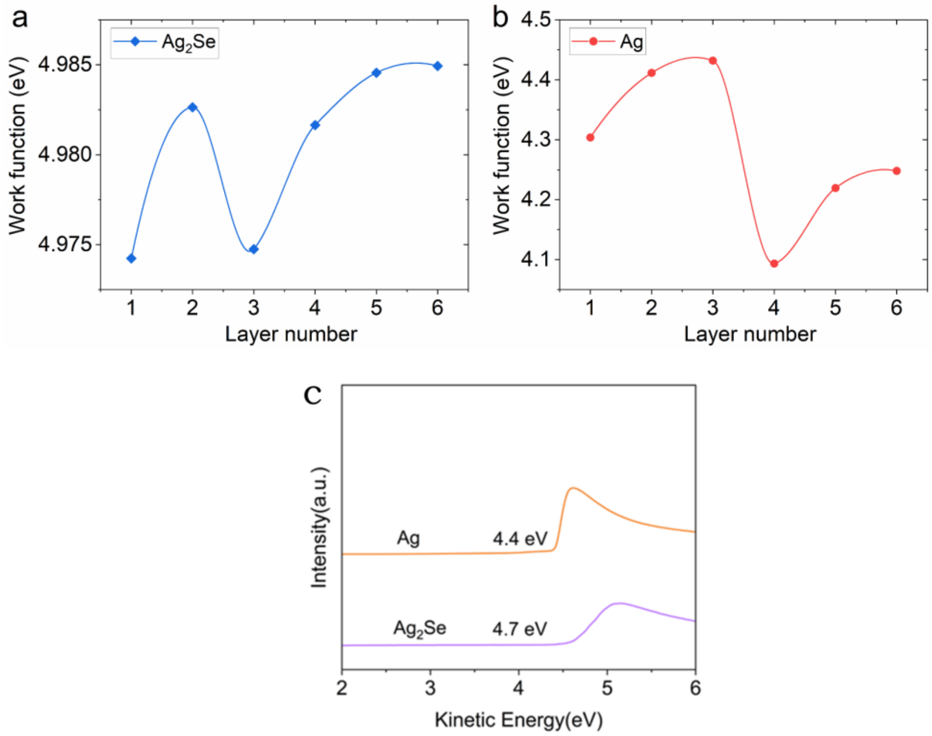

Supplementary Figure 9| Work function of (**a**) Ag_2Se and (**b**) Ag as a function of unit-cell layer number. **c.** Ultraviolet photoelectron spectroscopy (UPS) of secondary electron cutoff region of inkjet-printed Ag and Ag_2Se film.

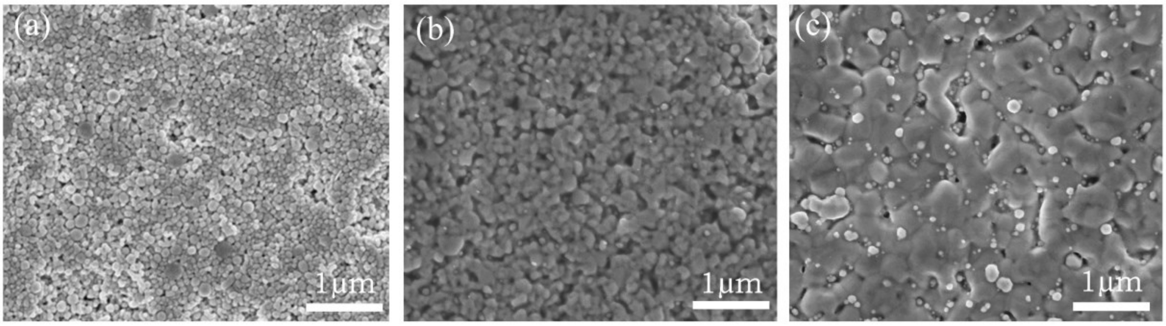

Supplementary Figure 10| SEM images of Ag_2Se/15%Ag samples: (a) as-printed, (b) film annealed at 573 K, (c) film annealed at 723 K.

Table S5. Room-temperature thermoelectric properties of Ag_2Se/15%Ag films annealed at different temperature.

Annealing temperature (K)	σ (S/cm)	α (μV/K)	PF (μW/mK^2)	n_H (cm^{-3})	μ_H (cm^2/Vs)
573	1565.2	-54.4	463.2	$6.28*10^{19}$	200.5
723	1040.2	-92.5	889.0	$1.63*10^{19}$	403.5

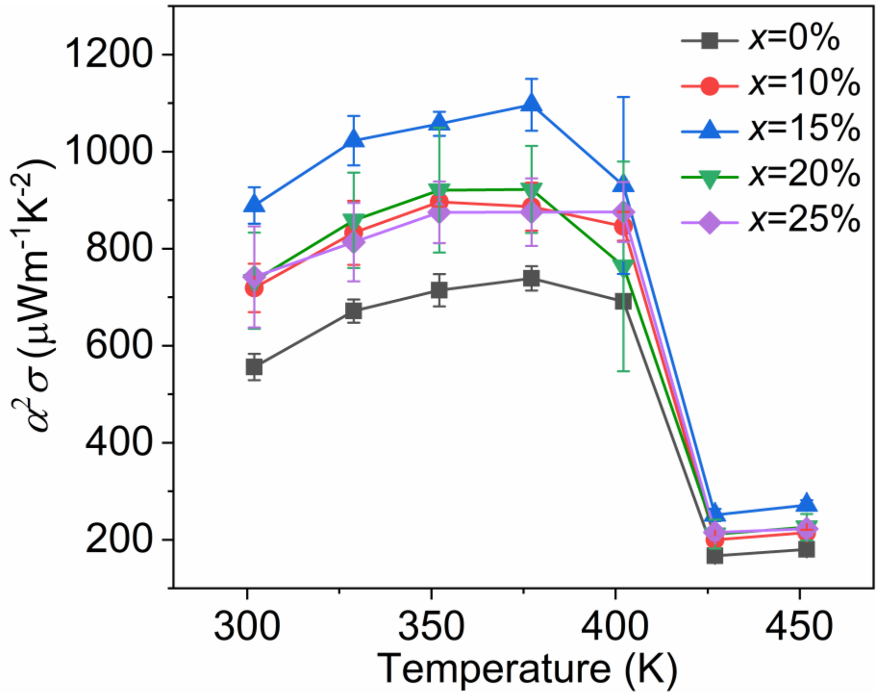

Supplementary Figure 11| Temperature-dependent power factor.

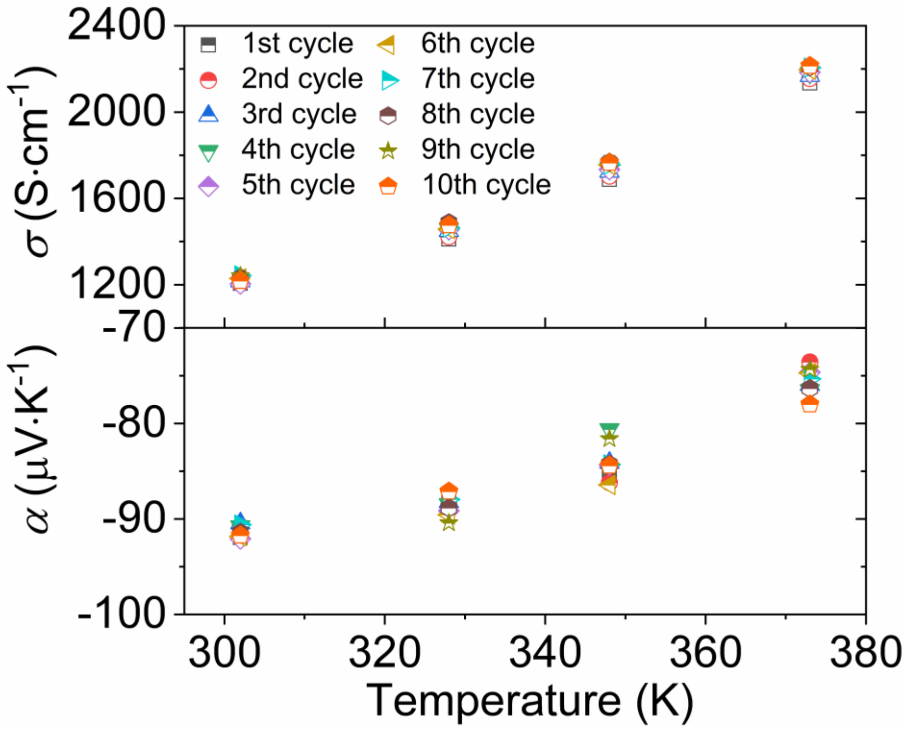

Supplementary Figure 12| Thermal cycling performance of the inkjet-printed Ag_2Se/15%Ag composite film.

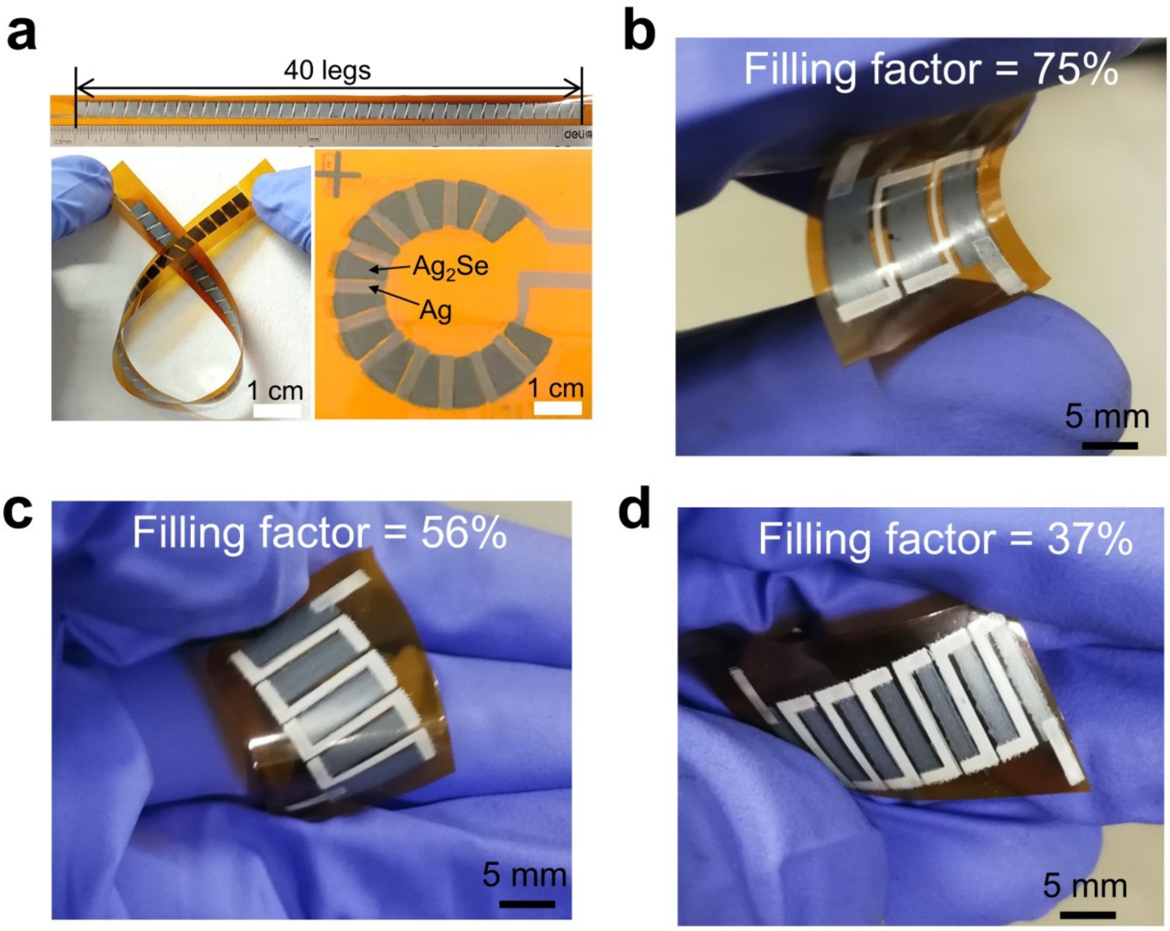

Supplementary Figure 13| a, Digital photographs of several fully inkjet-printed Ag_2Se-based flexible devices: a device consisting of 40 Ag_2Se legs; a ring-like device. **b-d,** Fully inkjet-printed Ag_2Se-based flexible thermoelectric devices with different filling factors in order to maintain a desired temperature gradient.

Supplementary Table S6| Dimensional parameters of five Ag_2Se-based flexible devices with the same aspect ratio.

Name	Leg length (mm)	Leg width (mm)	Leg thickness (μm)	Leg gap (mm)	Leg cross-sectional area (mm^2)	Leg aspect ratio (μm^{-1})	Device cross-sectional area (mm^2)
Device 1#	10.50	4	1.15	1.1	4.60E-03	2.28	4.57E-02
Device 2#	5.25	2	1.15	1.1	2.30E-03	2.28	2.73E-02
Device 3#	2.63	1	1.15	1.1	1.15E-03	2.28	1.81E-02
Device 4#	1.31	0.5	1.15	1.1	5.75E-04	2.28	1.35E-02
Device 5#	0.66	0.25	1.15	1.1	2.88E-04	2.28	1.12E-02

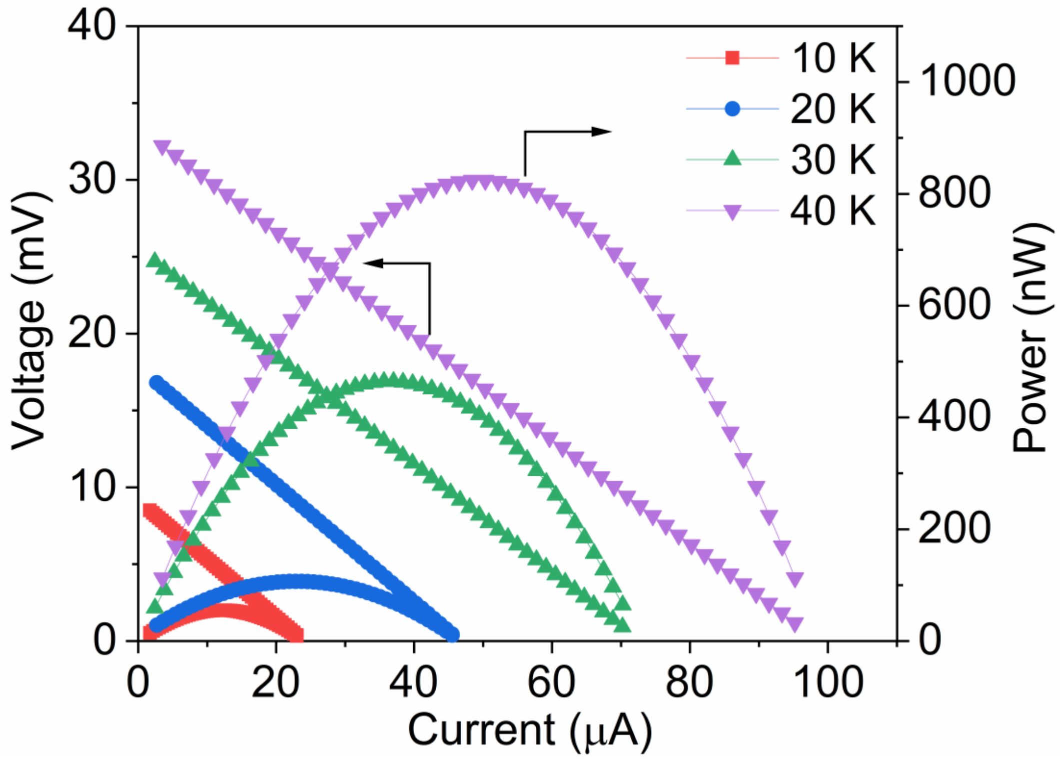

Supplementary Figure 14| Output voltage and power of Device #1 as a function of current at different temperature gradients.

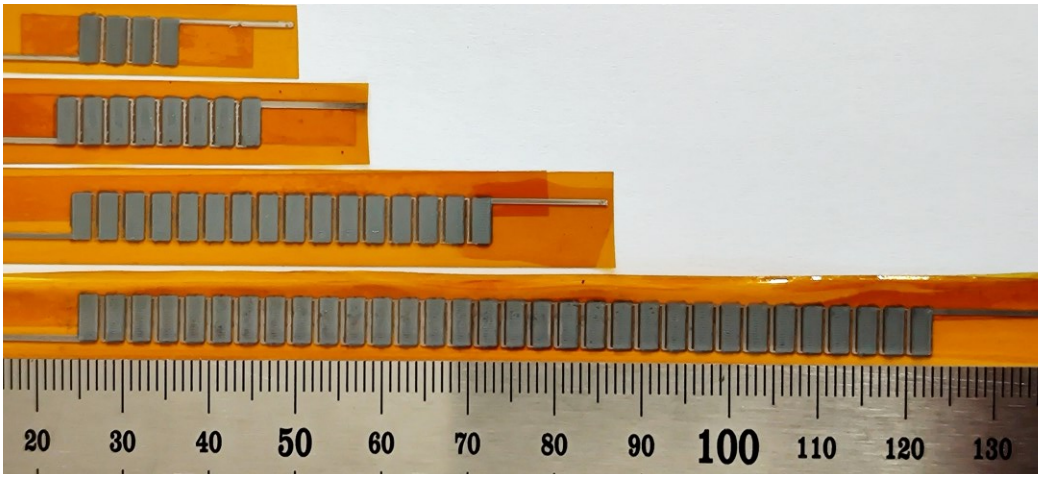

Supplementary Figure 15| Digital photograph of fully inkjet-printed Ag_2Se-based flexible device consisting of different number of Ag_2Se legs.

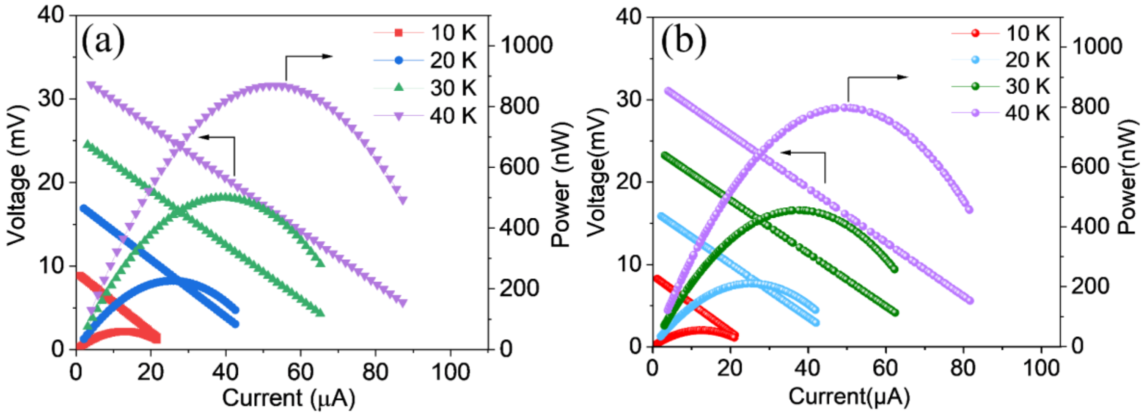

Supplementary Figure 16| Output voltage and power as a function of current at different temperature gradients: **a,** before, and **b,** after bending at a bending radius of 4 mm for 1000 times.

Table S7. Comparison of power density of a device subjected to bending for 1000 times.

$\triangle T$ (K)	Normalized power before bending ($\mu Wcm^{-2}K^{-2}$)	Normalized power after bending ($\mu Wcm^{-2}K^{-2}$)	Amount of degradation (%)
40	2.01	1.83	9.0
30	1.15	1.04	9.6
20	0.52	0.48	7.7
10	0.14	0.13	7.1

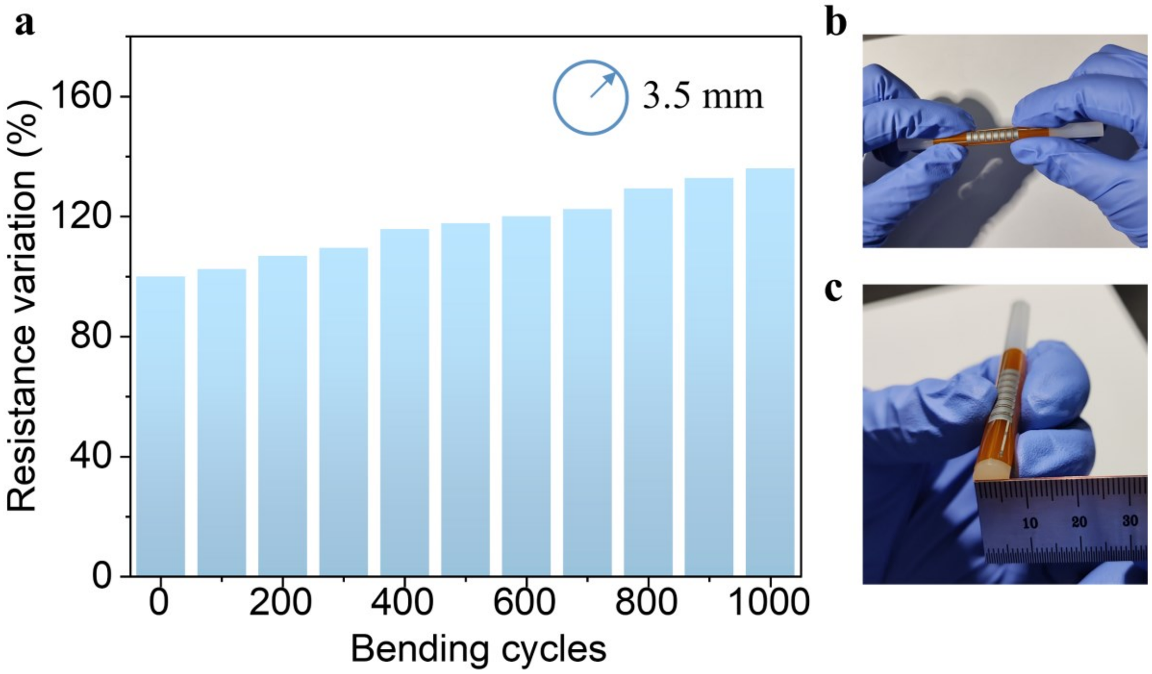

Supplementary Figure 17| a, Change of the device resistance after continuously repeated bending at a bending radius of 3.5 mm. **b**, and **c**, Photographs of bending tests.

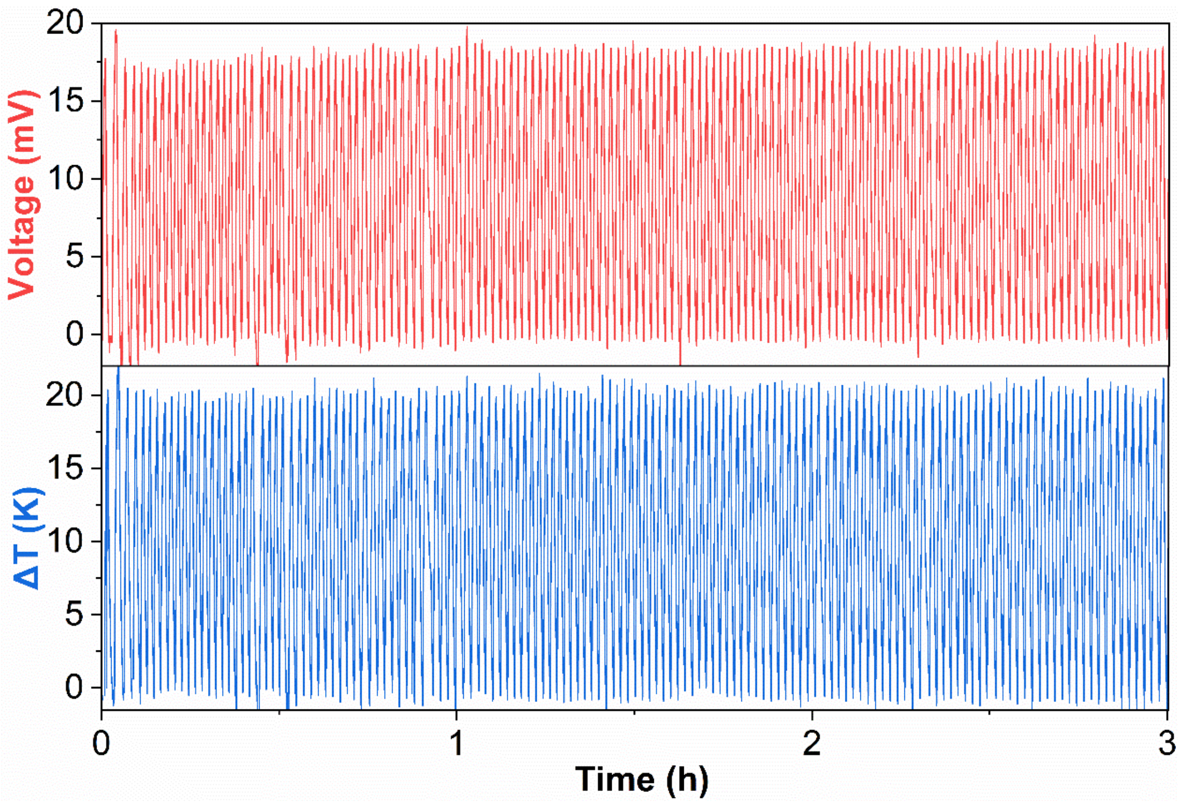

Supplementary Figure 18| Thermal stability of a fully inkjet-printed Ag_2Se-based flexible thermoelectric device. Change of temperature difference and voltage during heating-cooling cycles. It takes about 80 seconds for one cycle, and over 130 cycles were measured in total. The test was carried out in air.

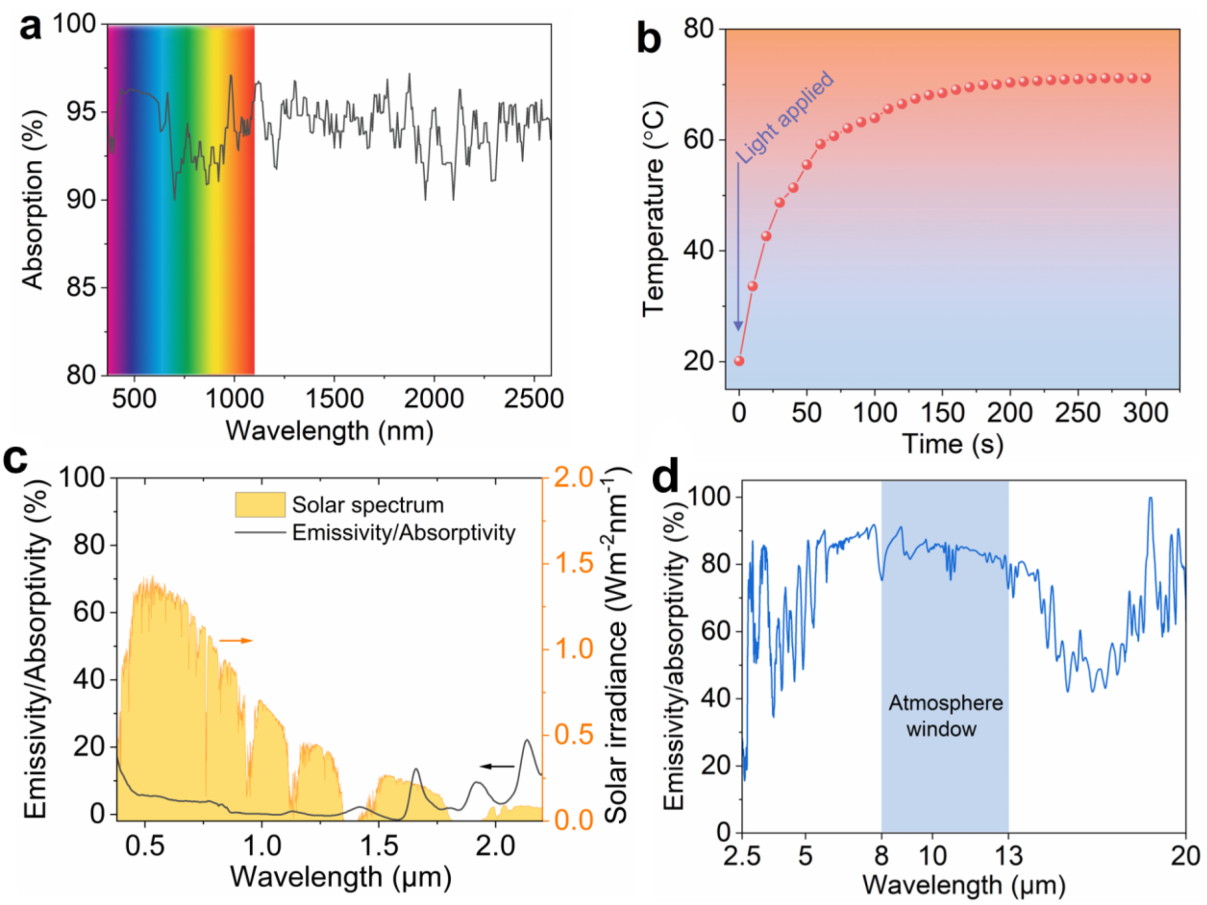

Supplementary Figure 19| a, Absorptivity of solar thermal material in visible light and near infrared spectra. **b**, Temperature change of solar thermal material under 1 sun illumination. **c**, Absorptivity of radiative cooling material in visible light and near infrared spectra. **d**, Emissivity of radiative cooling material in infrared spectrum.

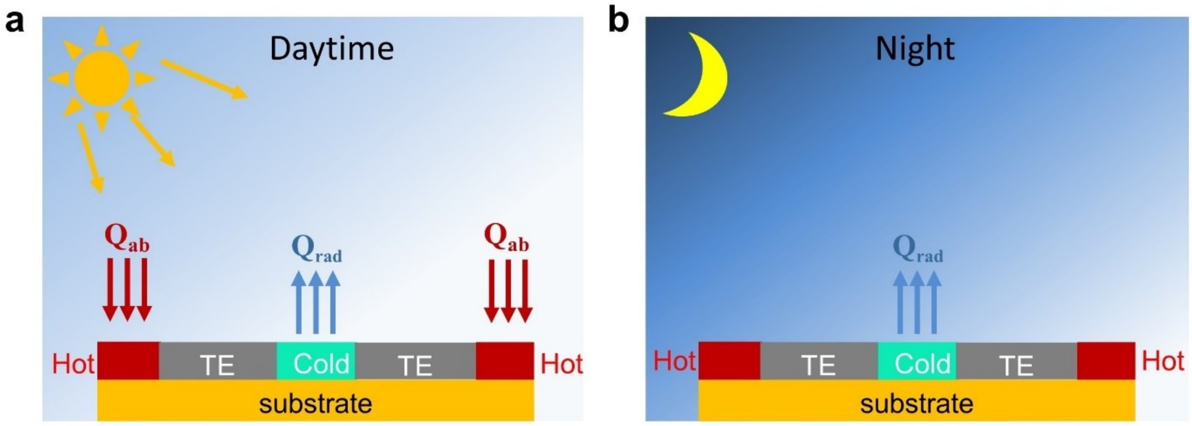

Supplementary Figure 20| Working schematic of a solar thermal/thermoelectric/radiative cooling (STR) hybrid device for 24-hour continuous power generation. **a**, At daytime. **b**, At night.

During the daytime, the photothermal material absorbs solar energy (Q_{ab}) and converts it into heat. The rise in temperature makes the photothermal material to be the hot side. At the same time, the radiative cooling material reflects the sunlight (Q_{rad}) and prevents the temperature in the central area of the device from rising, while the thermal insulation material inhibits the transfer of heat from the outer edge of the thermoelectric devices to the inner area. As a result, the inner edges of thermoelectric devices are kept at a lower temperature. A ΔT can thus be built up between the two ends of TE legs, allowing the thermoelectric devices to generate electricity.

At night, the temperature at the outer edge is close to the ambient temperature, while the temperature at the inner edge is lower due to the radiative cooling effect. This also allows the thermoelectric devices to build up a ΔT to generate electricity. The great advantage of our STR hybrid device is that it can generate electricity continuously for 24 hours with a constant direction of temperature gradient, avoiding the need to reverse the positive and negative terminals and thus requiring no maintenance.

Supplementary Note 1

In general, the inverse Ohnesorge number Z can be used to determine the jettability of the inks,[1] which is defined by:

$$Z = \frac{1}{oh} = \frac{Re}{\sqrt{We}} = \frac{\sqrt{\gamma \rho d}}{\eta}$$

Here, where ρ, η, and γ represent density (in kg/m^3), viscosity and surface tension of the ink, and d is the nozzle diameter. Oh is the Ohnesorge number. Re is the Reynolds number, and We is the Weber number, which can be calculated by:

$$Re = \frac{\rho v d}{\eta}$$

$$We = \frac{\rho v^2 d}{\gamma}$$

where v is the drop velocity after ejection from the nozzles.[1]

Supplementary Note 2

The Seebeck coefficient of thermoelectric materials can be expressed as follows according to the parabolic band structure for simplicity:[2]

$$S = \frac{8 \cdot \pi^2 \cdot k_{\mathrm{B}}^2}{3 \cdot e \cdot h^2} \cdot m^* \cdot T \cdot \left(\frac{\pi}{3n}\right)^{\frac{2}{3}} \cdot (r+1)$$

where k_{B}, e, h, m^*, n, μ, and r are the Boltzmann constant, electron charge, Planck constant, effective mass, carrier concentration, carrier mobility, and scattering parameter, respectively.

Supplementary Note 3

The dotted lines in Fig. 3d of main text are Seebeck coefficient as a function of carrier concentration calculated assuming a parabolic density of states and a simple power-law dependence of the relaxation time:[3]

$$\alpha = \pm \frac{k_B}{e} \left[\frac{\left(r+\frac{5}{2}\right) F_{r+3/2}(\zeta^*)}{\left(r+\frac{3}{2}\right) F_{r+1/2}(\zeta^*)} - \zeta^* \right]$$

$$n = \frac{N_V^{2/3}}{2\pi^2} \left(\frac{2 k_B T m_d}{\hbar^2}\right)^{3/2} F_{1/2}(\zeta^*)$$

where k_B is Boltzmann's constant, e is the electronic charge, $\zeta^*=E_F/(k_BT)$ is the reduced Fermi energy, m_d is the density of states effective mass, N_V is the number of degenerate valleys, r is the energy-dependent relaxation time exponent and F_s is the Fermi integral given by

$$F_s(\zeta^*) = \int_0^\infty x^s[exp(x - \zeta^*) + 1]^{-1}dx$$

Supplementary Note 4

The open-circuit voltage (V_{oc}) of a thermoelectric device can be theoretically calculated using the following equation:

$$V_{oc} = N \times |\alpha| \times \Delta T$$

N is the number of thermoelectric legs, $|\alpha|$ is absolute Seebeck coefficient, and ΔT is the temperature gradient.

The maximum output power (P_{max}) can be calculated using the following equation:

$$P_{max} = \frac{{V_{oc}}^2}{4R_{in}} = \frac{N\sigma\alpha^2\Delta T^2 A}{4l}$$

R_{in} is the internal resistance of the device. l is the length and A is the cross-sectional area of the thermoelectric leg. l/A stands for the aspect ratio.

Supplementary Methods

Materials

Selenium dioxide (SeO_2, ≥99.9%) and oleic acid (OA, 90%) were obtained from Sigma-Aldrich. Polyvinyl pyrrolidone (PVP K-30, average MW=40 000) were supplied by Adamas. Silver nitrate ($AgNO_3$, analytical grade), dimethyl formamide (DMF, analytical grade), ethanol (analytical grade), acetone (analytical grade) and methanol (analytical grade) were purchased from Sinopharm Chemical Reagent. All chemicals were used as purchased without further purification.

Synthesis of Ag_2Se powders

Ag_2Se nano-powders were synthesized by a solvothermal method modified based on a previous report.[4] In a typical synthetic procedure, 1g of PVP was first added into 40 mL of DMF with magnetic stirring for 8 minutes, followed by adding 0.052 g of SeO_2 and 0.164 g of $AgNO_3$.

Then, 20 mL of OA was added. The mixture was then transferred into a Teflon-lined stainless-steel autoclave (100 mL) and heated in an oven at 120 °C for 12 h. After the reaction was naturally cooled to room temperature, the precipitates were collected by centrifugation at 11000 rpm for 5 minutes and then washed with ethanol, acetone, methanol, and ethanol. The collected product was dispensed with sonication in 10 mL of ethanol for 2 h to form a Ag_2Se-based ink at a concentration of 10 $mg \cdot mL^{-1}$.

Synthesis of solar thermal and radiative cooling materials

Photothermal material was prepared by mixing 27 g carbon black (Aladdin, 99.9%) with 3 g 1-methoxy-2-propanol acetate (PGMEA, Aladdin, 99.9%) solvent. After adding 0.5 wt% PVP, the mixture was sand-milled at 2600 rpm for 8 h, with a break of 30 min for every 2h. A stable slurry was obtained by filtering the mixture through 200 mesh gauze. At the same time, a precursor solution was prepared by using 8 g pentaerythritol triacrylate (PETA, Aladdin, 99.9%) and an aliphatic urethane acrylate (AgiSyn 230A2, DSM-AGI, Taiwan) in a mass ratio of 8:2 and stirred at 500 rpm for 30 min. The slurry and the precursor solution were then mixed at 3:1 and stirred for another 30 min. The final slurry can be brushed on the desired position with a brush and dried at 50°C for 5 min. To prepare the radiative cooling material, 1 g SiO_2 microspheres with the diameter of 5 μm were placed between two PDMS substrates and then rubbed against each other for 30 s until the SiO_2 microspheres were tightly packed. Then, a solution consisting of SiO_2 spheres (200 nm), acetone and PMMA in a mass ratio of 1:10:1 was prepared and stirred at 50°C for 10 h. 5 g solution was then filled into the tightly packed SiO_2 microspheres. After complete evaporation of the solvent, the PDMS was completely removed. The SiO_2 was then dissolved by using HF acid, resulting in a PMMA radiative cooling film with the thickness of 5 μm.

Supplementary References

1. Majee, S. *et al.* Low temperature chemical sintering of inkjet-printed Zn nanoparticles for highly conductive flexible electronic components. *npj Flex Electron* **5**, 14 (2021).
2. Lu, X. *et al.* High-efficiency thermoelectric power generation enabled by homogeneous incorporation of MXene in $(Bi,Sb)_2Te_3$ matrix. *Adv. Energy Mater.* **10**, 1902986 (2020).
3. Sumithra, S. *et al.* Enhancement in thermoelectric figure of merit in nanostructured Bi_2Te_3

with semimetal nanoinclusions. *Adv. Energy Mater.* **1**, 1141 (2011).

4. Wang, J. *et al.* Tetragonal-Orthorhombic-Cubic phase transitions in Ag_2Se nanocrystals. *Chem. Mater.* **26**, 5647-5653 (2014).
